# Low Multiplication Value of Absolute Monocyte Count and Absolute Lymphocyte Count at Diagnosis May Predict Poor Prognosis in Neuroblastoma

**DOI:** 10.3389/fonc.2020.572413

**Published:** 2020-10-02

**Authors:** Akihiro Tamura, Shotaro Inoue, Takeshi Mori, Jun Noguchi, Sayaka Nakamura, Atsuro Saito, Aiko Kozaki, Toshiaki Ishida, Kay Sadaoka, Daiichiro Hasegawa, Yoshiyuki Kosaka, Masanori Miyanishi

**Affiliations:** ^1^Department of Hematology and Oncology, Kobe Children’s Hospital, Kobe, Japan; ^2^Laboratory for Organismal Patterning, RIKEN Center for Biosystems Dynamics Research, Kobe, Japan

**Keywords:** Neuroblastoma, monocyte, lymphocyte, Blood cell counts, prognosis

## Abstract

Despite the growing evidences that immune dysfunction contributes to tumor progression, the prognostic value in patients with neuroblastoma regarding circulating immune blood cell counts has not been well characterized. To answer this, we conducted a retrospective study to evaluate the prognostic value of the circulating immune cell counts at diagnosis in a cohort of 55 patients with neuroblastoma. Based on a novel index by multiplying the absolute monocyte count (AMC)/μl and absolute lymphocyte count (ALC)/μl, we sub-grouped patients with AMC × ALC ≥ 1 × 10^6^ (/μl)^2^ as high group and patients with AMC × ALC < 1 × 10^6^ (/μl)^2^ as low group. In the entire cohort, the 4-year progression-free survival (PFS), and overall survival (OS) for high group (*n* = 38) vs low group (*n* = 17) was 81.7% (95%CI; 63.6–91.3%) and 90.7% (95%CI; 73.8–96.9%) vs 31.7% (11.6–54.1%) and 56.5% (29.7–76.4%; *p* < 0.001 for PFS and *p* = 0.015 for OS), respectively, suggesting that a low AMC × ALC is associated with poor prognosis. In the subgroup analysis for high-risk patients, the 4-year PFS and OS for high group (*n* = 17) vs low group (*n* = 13) was 59.8% (31.2–79.7%) and 79.8% (49.4–93.0%) vs 8.5% (0.5–31.7%) and 42.0% (15.4–66.8%; *p* < 0.001 for PFS and *p* = 0.089 for OS), respectively. Our data demonstrate that AMC × ALC at diagnosis is a cost-effective and easily measurable biomarker for predicting prognosis in neuroblastoma.

## Introduction

Neuroblastoma is the most common type of extracranial solid tumor and is the leading cause of cancer-related death in children (1). Despite recent therapeutic advances, the poor prognosis of high-risk neuroblastoma has not been improved (2). Given the accumulating clinical evidences that impaired immune function contributes to tumor progression, we believe that understanding the immunological mechanisms behind this phenomenon may provide clues to new treatment strategies for high-risk neuroblastoma (3).

While the clinical impact of T-cell dysfunction has been widely examined, numerous studies examining the effect of circulating blood cell counts, including lymphocytes, neutrophils, and monocytes, showed that their results varied widely depending on the types and cohorts of cancers (4–9).

Recent clinical data suggest that monocytes may also play an essential role in controlling cancer progression (10, 11).

However, the impact of the composition of circulating immune cells on the prognosis has not been elucidated in neuroblastoma. In this study, we aimed to clarify the impact of the patient’s immune status on the prognosis of pediatric neuroblastoma by retrospectively comparing circulating immune cells at the time of the initial presentation and clinical outcomes.

## Materials and Methods

We retrospectively analyzed the prognostic value of the circulating immune cell counts at the initial presentation in a cohort of 55 patients with neuroblastoma diagnosed at Kobe Children’s Hospital (Kobe, Japan) between January 2006 and January 2020. Complete blood counts were obtained using an automatic blood analyzer (XN-2000 or XS-500i, Sysmex, Kobe, Japan), and a differential count was performed manually in most (52 of 55) cases. All complete blood counts were performed prior to the initiation of surgical resection or chemotherapy. Continuous variables were dichotomized based on the optimized cut-off values determined by receiver operating characteristic curve (ROC) analysis to predict progression-free survival (PFS). We created a novel index by multiplying the absolute monocyte count (AMC)/μl and absolute lymphocyte count (ALC)/μl at the initial presentation (AMC × ALC; /μl)^2^. PFS was defined as the time from the date of diagnosis to the date of relapse or progression. Overall survival (OS) was defined as the time from the date of diagnosis to the date of death. PFS and OS were analyzed by the Kaplan–Meier method and log-rank test, and the observation period was censored at 8 years.

Mann–Whitney *U* test was used to compare differences between the two groups. Kruskal–Wallis test was used to compare differences in more than two groups. Fisher’s exact test was used to compare the distribution of categorical variables between groups. Univariate Cox regression model was used to estimate the hazard ratio (HR) for relapse/progression or death. Significant prognostic factors in the univariate analysis (AMC × ALC < 1 × 10^6^ (/μl)^2^, Hb < 10 *g*/dl, and ferritin ≥ 350 ng/ml) were included in the multivariate analyses using the Cox proportional hazards model. A value *p* < 0.05 was considered as statistically significant. All statistical analyses were performed using EZR (Saitama Medical Center, Jichi Medical University, Saitama, Japan), a graphical user interface for R (The R Foundation for Statistical Computing, Vienna, Austria) (12). The retrospective review of medical records was approved by the institutional review board of the Kobe Children’s Hospital. This research was conducted in accordance with the Declaration of Helsinki.

## Results

The cohort includes a total of 55 children with neuroblastoma with a median observation period of 5.4 years (range; 0.2–8.0 years), comprising of 30 high-risk (HR), 6 intermediate-risk (IR), and 19 low-risk (LR) patients based on the risk classification of the children’s oncology group (COG) (13). To identify prognostic factors for HR patients, we compared baseline laboratory data between the patients for each COG risk classification (Table 1). The white blood cell count (WBC), AMC, and absolute neutrophil count (ANC) did not differ significantly among risk groups. However, the ALC (mean ± SD) was markedly lower in HR patients (HR; 3,477 ± 1,814/μl, IR; 5,034 ± 1,721/μl, and LR; 4,595 ± 1,641/μl; *p* = 0.042), suggesting that low ALC is a potential feature for high-risk neuroblastoma patients. The hemoglobin (Hb) level was significantly lower in HR patients, while the platelet count (Plt) was not significantly affected. As expected, neuron-specific enolase (NSE), lactate dehydrogenase (LDH), C-reactive protein (CRP), and ferritin levels were higher in HR patients. 4-year PFS and OS in each COG risk group was 36.9% (95% CI; 19.3–54.7%) and 62.5% (95% CI; 41.4–77.8%) for HR, 100 and 100% for IR, and 100 and 100% for LR, respectively.

**TABLE 1:** Baseline laboratory data of the patients in each risk groups.

	High risk (*n* = 30)	Intermediate risk (*n* = 6)	Low risk (*n* = 19)	*p* value
WBC (/μl)	9,0973,637	9,1002,875	9,3262,991	0.956
AMC (/μl)	524350	602347	608435	0.596
ALC (/μl)	3,4471,814	5,0341,721	4,5951,641	0.042
ANC (/μl)	4,9732,866	3,2021,928	3,6952,002	0.131
Hb (g/dl)	9.52.1	10.81.4	12.42.1	<0.001
Plt (×10^4^/μl)	31.214.8	36.211.5	38.911.0	0.052
NSE (ng/ml)	452388	8033	5372	<0.001
VMA (μg/mg Cre)	130144	194122	153302	0.163
HVA (μg/mg Cre)	174131	17191	122214	0.007
LDH (U/l)	1,4881,466	31864	323150	<0.001
CRP (mg/dl)	5.67.0	0.10.1	0.30.1	<0.001
Ferritin (ng/ml)	244208	3326	6569	<0.001

*ALC, absolute lymphocyte counts; AMC, absolute monocyte count; ANC, absolute neutrophil count; Cre, creatinine; CRP, C-reactive protein; Hb, hemoglobin; HVA, homovanillic acid; LDH, lactate dehydrogenase; NSE, neuron-specific enolase; Plt, platelet count; VMA, vanillylmandelic acid; and WBC, white blood cell count.*

We next aimed to elucidate the association between blood cell counts and clinical outcomes. Due to the fluctuation of blood cell count after therapy, we could not identify any prognostic significance of blood cell count at any time point after treatment (data not shown).

**FIGURE 1:**
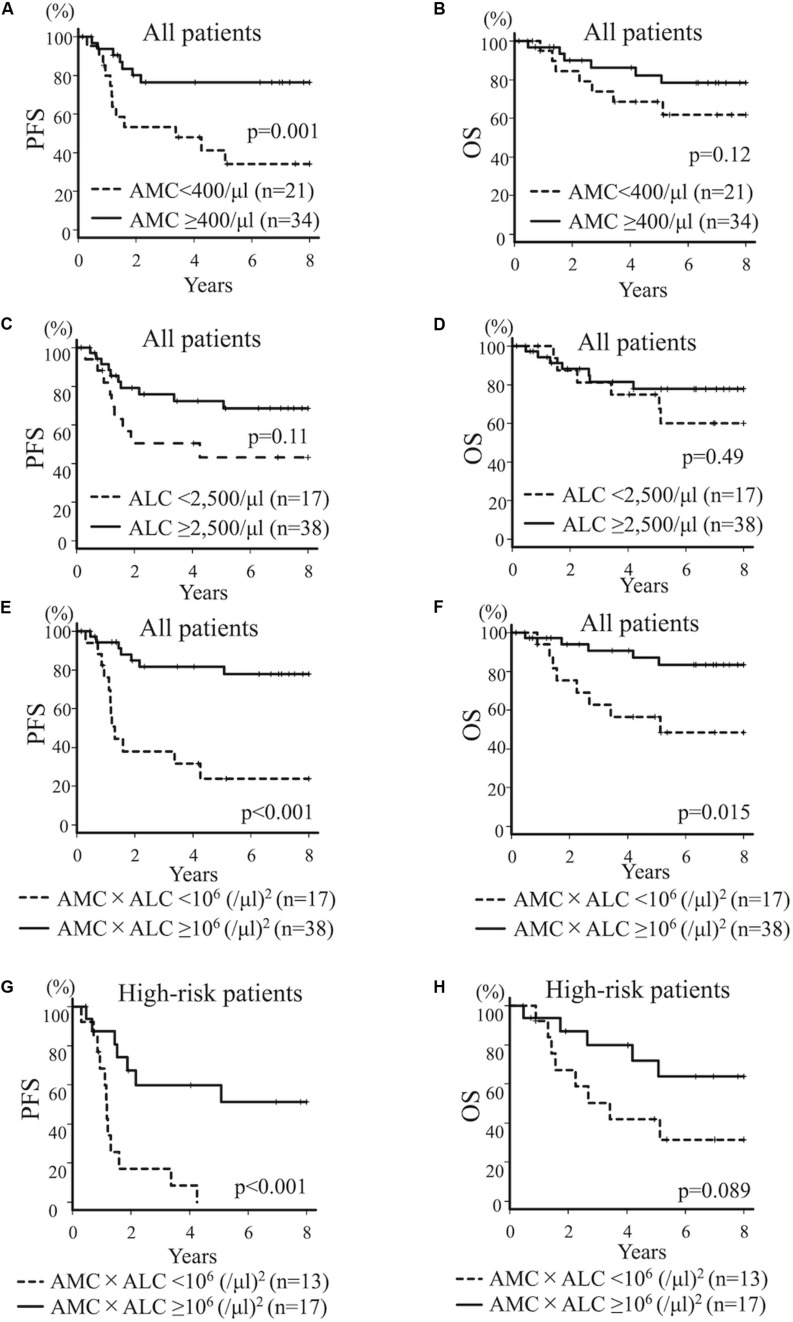
Kaplan–Meier curves for all patients **(A–F)** and high-risk patients **(G,H)** with neuroblastoma. PFS **(A)** and OS **(B)** for all patients with neuroblastoma dichotomized by AMC at diagnosis (AMC ≥ 400/μl vs AMC < 400/μl). PFS **(C)** and OS **(D)** for all patients with neuroblastoma dichotomized by ALC at diagnosis (ALC ≥ 2,500/μl vs ALC < 2,500/μl). PFS **(E)** and OS **(F)** for all patients with neuroblastoma dichotomized by AMC × ALC at diagnosis [AMC × ALC ≥ 1 × 10^6^ (/μl)^2^ vs AMC × ALC < 1 × 10^6^ (/μl)^2^]. PFS **(G)** and OS **(H)** for patients with high-risk neuroblastoma dichotomized by AMC × ALC at diagnosis [AMC × ALC ≥ 1 × 10^6^ (/μl)^2^ vs AMC × ALC < 1 × 10^6^ (/μl)^2^].

Then, using the pre-therapeutic blood cell count, we dichotomized the entire cohort according to the levels of AMC, ALC, and ANC at diagnosis. The cut off values (400/μl for AMC, 2,500/μl for ALC, and 4,000/μl for ANC) were derived from the ROC to predict PFS. 4-year PFS and OS for all patients with AMC ≥ 400/μl (*n* = 34) vs AMC < 400/μl (*n* = 21) was 76.4% (95% CI; 56.7–88.0%) and 86.2% (95% CI; 67.2–94.6%) vs 47.9% (95% CI; 24.9–67.8%) and 68.6% (95% CI; 43.0–84.5%; *p* = 0.001 for PFS and *p* = 0.12 for OS), respectively, suggesting that lower AMC is associated with poorer PFS (Figures 1A,B). 4-year PFS and OS for all patients with ALC ≥ 2,500/μl (*n* = 38) vs ALC < 2,500/μl (*n* = 17) was 72.4% (95% CI;53.5–84.7%) and 81.3% (95% CI; 62.9–91.2%) vs 50.4% (95% CI; 24.9–71.4%) and 75.0% (95% CI; 46.3–89.8%; *p* = 0.11 for PFS and *p* = 0.49 for OS; Figures 1C,D). Although not statistically significant, patients with poorer prognosis tended to have a lower ALC. 4-year PFS and OS for all patients with ANC ≥ 4,000/μl (*n* = 26) vs ANC < 4,000/μl (*n* = 29) was 67.3% (95% CI; 45.1–82.2%) and 74.6% (95% CI; 51.9–87.8%) vs 63.7% (95% CI; 41.7–79.3%) and 83.7% (62.2–93.6%; *p* = 0.89 for PFS and *p* = 0.36 for OS), respectively; not statistically significant. Based on these results, we introduced a novel prognostic index by multiplying the AMC/μl and ALC/μl to further improve the prediction of prognosis, and sub-grouped patients with AMC × ALC ≥ 1 × 10^6^ (/μl)^2^ as high group and patients with AMC × ALC < 1 × 10^6^ (/μl)^2^ as low group.

Four-year PFS and OS for all patients with AMC × ALC high group (*n* = 38) vs AMC × ALC low group (*n* = 17) was 81.7% (95%CI; 63.6–91.3%) and 90.7% (95%CI; 73.8–96.9%) vs 31.7% (11.6–54.1%) and 56.5% (29.7–76.4%; *p* < 0.001 for PFS *p* = 0.015 for OS), respectively, suggesting that low AMC × ALC is associated with poorer prognosis (Figures 1E,F). The HR (AMC × ALC low group compared to AMC × ALC high group) for relapse/progression and death was 5.82 (95%CI; 2.26–14.97, *p* < 0.001) and 4.02 (95%CI; 1.31–12.32, *p* = 0.015) among all patients, respectively. These results demonstrate the prognostic significance of low AMC × ALC at diagnosis for children with neuroblastoma.

**FIGURE 2:**
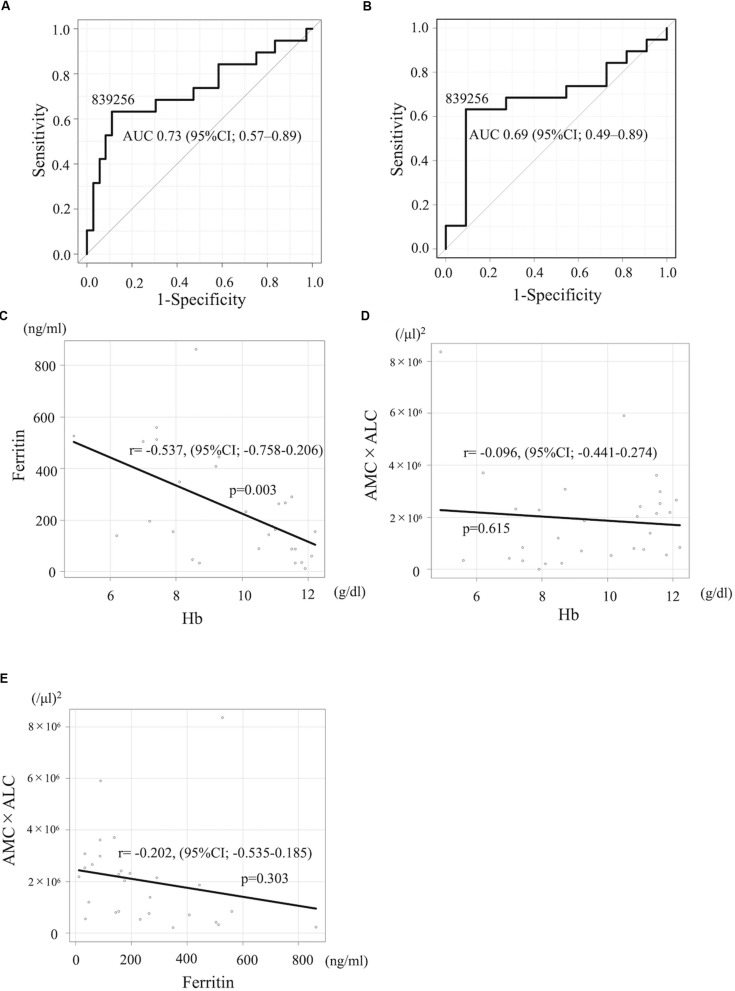
**(A,B)** The ROC curve of the AMC × ALC for relapse/progression for all patients and HR neuroblastoma patients. **(C–E)** Pearson product moment correlation coefficient between Hb and ferritin levels **(C)**, Hb and AMC × ALC **(D)**, and ferritin and AMC × ALC **(E)**.

The development of novel biomarkers to predict the risk of relapse before starting the therapy can lead to an optimal strategy of treatment for neuroblastoma, especially with HR patients. We next evaluated the prognostic impact of these indices limited for HR patients. 4-year PFS and OS for high-risk patients with AMC ≥ 400/μl (*n* = 16) vs AMC < 400/μl (*n* = 14) was 49.5% (95%CI; 22.0%–72.2%) and 71.1% (95%CI; 39.8–88.1%) vs 23.6% (95%CI; 5.7–48.2%) and 54.2% (95%CI; 25.0–76.2%; *p* = 0.032 for PFS and *p* = 0.66 for OS), respectively, suggesting that lower AMC is associated with poorer PFS. 4-year PFS and OS for HR patients with ALC ≥ 2,500/μl (*n* = 17) vs ALC < 2,500/μl (*n* = 13) was 38.5% (95%CI; 14.8–62.0%) and 58.9% (95%CI; 30.2–79.2%) vs 33.8% (95%CI; 10.5–59.4%) and 66.7% (95%CI; 33.7–86.0%; *p* = 0.59 for PFS and *p* = 0.92 for OS), respectively; not statistically significant. 4-year PFS and OS for HR patients with AMC × ALC high group (*n* = 17) vs AMC × ALC low group (*n* = 13) was 59.8% (31.2–79.7%) and 79.8% (49.4–93.0%) vs 8.5% (0.5–31.7%) and 42.0% (15.4–66.8%; *p* < 0.001 for PFS and *p* = 0.089 for OS), respectively, (Figures 1G,H). The HR (AMC × ALC low group compared to AMC × ALC high group) for PFS and OS was 5.18 (95% CI; 1.88–14.32, *p* < 0.001) and 2.56 (95% CI; 0.83–7.88, *p* = 0.089) among HR patients, respectively. These results indicated that the AMC × ALC < 1 × 10^6^ (/μl)^2^ at the initial presentation is associated with poorer PFS, even among HR patients, although the prognostic significance to predict poorer OS did not reach statistical significance. The area under the ROC curve of the AMC × ALC for relapse/progression for all patients and HR patients was 0.73 (95%CI; 0.57–0.89; Figure 2A) and 0.69 (95%CI; 0.49–0.89; Figure 2B), respectively.

Next, to examine if the AMC × ALC can work as an independent prognostic factor for high-risk neuroblastoma, we conducted univariate and multivariate analysis, including other known prognostic markers. In the univariate analysis, the AMC < 400/μl, AMC × ALC < 1 × 10^6^ (/μl)^2^, Hb < 10 *g*/dl, and ferritin ≥ 350 ng/ml were significant risk factors for poorer PFS among patients with high-risk neuroblastoma (Table 2). In contrast, Plt, NSE, vanillylmandelic acid (VMA), homovanillic acid (HVA), LDH, CRP, and N-myc amplification, were not associated with poorer PFS among patients with high-risk neuroblastoma.

**TABLE 2:** Univariate analysis of the factors associated with prognosis in children with high-risk neuroblastoma.

Variables	Classification	*N*	PFS		OS	
				
			HR (95% CI)	*p* value	HR (95% CI)	*p* value
WBC	≥9,000/μl	15	1		1	
	<9,000/μl	15	2.01 (0.78–5.15)	0.147	1.46 (0.48–4.45)	0.512
AMC	≥400/μl	16	1		1	
	<400/μl	14	2.69 (1.05–6.88)	0.039	1.28 (0.43–3.81)	0.658
ALC	≥2,500/μl	17	1		1	
	<2,500/μl	13	1.28 (0.52–3.16)	0.591	0.94 (0.32–2.81)	0.916
ANC	≥4,000/μl	17	1		1	
	<4,000/μl	13	1.42 (0.57–3.52)	0.445	0.87 (0.29–2.59)	0.797
AMC × ALC	≥10^6^ (/μl)^2^	17	1		1	
	<10^6^ (/μl)^2^	13	5.18 (1.88–14.32)	<0.001	2.56 (0.83–7.88)	0.089
Hb	≥10 *g*/dl	15	1		1	
	<10 *g*/dl	15	3.02 (1.15–7.91)	0.025	3.15 (0.96–10.31)	0.057
Plt	≥34 × 10^4^/μl	12	1		1	
	<34 × 10^4^/μl	18	1.66 (0.63–4.39)	0.306	2.28 (0.62–8.29)	0.212
NSE	≥500 ng/ml	11	1		1	
	<500 ng/ml	19	0.90 (0.34–2.38)	0.830	0.63 (0.20–1.93)	0.416
VMA	≥120 μg/mg Cre	12	1		1	
	<120 μg/mg Cre	18	0.44 (0.18–1.10)	0.081	0.75 (0.25–2.24)	0.606
HVA	≥180 μg/mg Cre	12	1		1	
	<180 μg/mg Cre	18	0.77 (0.31–1.91)	0.579	1.16 (0.38–3.56)	0.793
LDH	≥1,200 U/l	11	1		1	
	<1,200 U/l	19	0.79 (0.31–2.03)	0.625	0.50 (0.17–1.52)	0.225
CRP	≥5 mg/dl	13	1		1	
	<5 mg/dl	17	0.60 (0.24–1.50)	0.273	0.32 (0.11–1.00)	0.050
Ferritin	<350 ng/ml	21	1		1	
	≥350 ng/ml	7	8.41 (2.24–31.57)	0.002	6.19 (1.94–19.72)	0.002
N-myc amplification	No	16	1		1	
	Yes	14	0.61 (0.24–1.55)	0.300	1.13 (0.38–3.36)	0.831

*ALC, absolute lymphocyte counts; AMC, absolute monocyte count; ANC, absolute neutrophil count; CI, confidence interval; CRP, C-reactive protein; Hb, hemoglobin; HR, hazard ratio; HVA, homovanillic acid; LDH, lactate dehydrogenase; NSE, neuron-specific enolase; OS, overall survival; PFS, progression-free survival; Plt, platelet count; VMA, vanillylmandelic acid; and WBC, white blood cell count.*

The baseline characteristics including age, gender, N-myc amplification, tumor cells percentage in the bone marrow, NSE, and year of diagnosis of patients with high-risk neuroblastoma dichotomized by AMC × ALC did not differ significantly between groups with exception of ferritin levels (Table 3). 4-year PFS and OS for high-risk patients with ferritin ≥ 350 ng/ml (*n* = 7) vs ferritin < 350 ng/ml (*n* = 21) was 0% and 17.1% (95%CI; 0.8–52.6%) vs 46.7% (95%CI; 23.5–67.0%) and 78.3% (95%CI; 51.9–91.3%; *p* < 0.001 for PFS and *p* < 0.001 for OS). To exclude the possibility that AMC × ALC is a confounding factor of Hb or ferritin levels, we next examined the correlations between AMC × ALC, Hb, and ferritin levels by Pearson product-moment correlation coefficient. Hb levels were inversely correlated with ferritin levels (Figure 2C). However, AMC × ALC was not significantly correlated with Hb or ferritin levels (Figures 2D–E). In the multivariate analysis, the AMC × ALC < 1 × 10^6^ (/μl)^2^ was the only independent risk factor for poorer PFS in patients with high-risk neuroblastoma (HR 3.97, 95%CI; 1.14–13.76, *p* = 0.030), in contrast to Hb < 10 *g*/dl (HR 1.76, 95%CI; 0.49–6.30, *p* = 0.383) and ferritin ≥ 350 ng/ml (HR 2.57, 95%CI; 0.52–12.78, and *p* = 0.248; Table 4).

**TABLE 3:** Baseline characteristics of patients with high-risk neuroblastoma dichotomized by AMC × ALC at diagnosis.

Variables	AMC × ALC < 10^6^ (/μl)^2^ (*n* = 13)	AMC × ALC ≥ 10^6^ (/μl)^2^ (*n* = 17)	*p* value
**Age**			
<18 months	2	2	1.000
≥18 months	11	15	
Gender			
Male	5	13	0.061
Female	8	4	
N-myc amplification			
Positive	4	10	0.159
Negative	9	7	
Neuroblastoma cells percentage in the bone marrow (%)	19.2 ± 29.8	9.0 ± 21.0	0.463
**Biochemical markers**			
NSE (ng/ml)	479 ± 435	432 ± 360	0.558
VMA (μg/mg Cre)	155 ± 156	110 ± 135	0.341
HVA (μg/mg Cre)	175 ± 123	172 ± 141	0.773
Hb (g/dl)	9.0 ± 2.0	9.8 ± 2.2	0.286
LDH (U/l)	1,387 ± 1,487	1,565 ± 1,490	0.660
CRP (mg/dl)	7.3 ± 9.5	4.3 ± 3.9	0.645
Ferritin (ng/ml)	366 ± 236	165 ± 145	0.020
**Year of diagnosis**			
2006–2010	3	6	0.889
2011–2015	7	7	
2016–2020	3	4	
Cord blood transplantation	6	3	0.123

*ALC, absolute lymphocyte counts; AMC, absolute monocyte count; Cre, creatinine; CRP, C-reactive protein; Hb, hemoglobin; HVA, homovanillic acid; LDH, lactate dehydrogenase; NSE, neuron-specific enolase; Plt, platelet count; and VMA, vanillylmandelic acid.*

**TABLE 4:** Multivariate analysis of the factors associated with prognosis in children with high-risk neuroblastoma.

Variables	Classification	*N*	PFS		OS	
				
			HR (95% CI)	*p* value	HR (95% CI)	*p* value
AMC × ALC	≥10^6^ (/μl)^2^	17	1		1	
	<10^6^ (/μl)^2^	13	3.97 (1.14–13.76)	0.030	1.12 (0.27–5.31)	0.813
Hb	≥10 *g*/dl	15	1		1	
	<10 *g*/dl	15	1.76 (0.49–6.30)	0.383	1.39 (0.25–7.66)	0.709
Ferritin	<350 ng/ml	21	1		1	
	≥350 ng/ml	7	2.57 (0.52–12.78)	0.248	4.40 (0.65–29.95)	0.130

*ALC, absolute lymphocyte counts; AMC, absolute monocyte count; CI, confidence interval; Hb, hemoglobin; HR, hazard ratio; OS, overall survival; and PFS, progression-free survival.*

## Discussion

High-risk neuroblastoma remains a refractory disease with poor prognosis despite recent medical advances. To date, numerous prognostic markers such as elevated serum ferritin, elevated LDH, and low Hb have been reported in patients with neuroblastoma (14–18). But these alterations are presumably induced by tumor progression such as bone marrow metastasis and are unlikely to be the targets for therapeutic development. Hence, the identification of new prognostic immune biomarkers and the development of optimal treatments using them is highly desirable.

In this study, we found that the multiplication of AMC and ALC at diagnosis (AMC × ALC) is a new prognostic indicator to identify patients with poor prognosis, based on an analysis of laboratory data from the entire cohort during the neuroblastoma treatment period. Further analysis using this novel prognostic measure in the same group revealed that low AMC × ALC at the diagnosis was significantly associated with poor prognosis, even when restricted to high-risk patients. These data suggest that pre-treatment immune status may have some effect on the antitumor effects of treatment. It is also important to note that the long-term prognosis of high-risk patients with low AMC × ALC at diagnosis is exceptionally low (4-year PFS 8.5%) during a median observation period of 5.4 years.

Previous reports have already shown that abnormal balance of cytotoxic T-cells/helper T-cells or Th1/Th2 subsets contributes to some cancer progression, including breast, colon, and liver cancer (19–21). In neuroblastoma, a decrease in regulatory T cells has been noted, but its correlation with prognosis has not been identified (22). On the other hand, our finding suggests that in addition to lymphocytes, monocytes and macrophages are actively involved in the clinical prognosis of neuroblastoma. Indeed, clinical studies showed that higher infltration of tumor-associated macrophages is associated with clinical aggressiveness in neuroblastoma (23, 24). Tumor-associated macrophages stimulate angiogenesis, increase tumor cell invasion, and suppress antitumor immunity (25). Therefore, targeting macrophages, such as anti-CSF1R antibodies, have emerged as attractive therapeutic approaches in various types of cancer (25). In addition, recent studies that C/EBPβ-dependent non-classical monocytes regulate cancer progression also support our results (10, 11, 26–28). In this cohort, most patients did not receive anti-GD2 antibody treatment because it is not approved in Japan. As an alternative, most of the relapsed cases received killer-cell immunoglobulin-like receptor-ligand mismatch cord blood transplantation in anticipation of a graft vs tumor effect (*n* = 6 in low AMC × ALC group and *n* = 3 in high AMC × ALC group; Table 3) (29, 30). This fact may be the reason why the OS did not reach a significant difference despite significantly worse PFS in high-risk patients with low AMC × ALC, suggesting the importance of immune cells in neuroblastoma progression.

To our knowledge, this is the first report of a predictive immune biomarker for the prognosis of patients with neuroblastoma, especially those at high risk. However, due to the relatively small sample size and short follow-up range of this single-center study, it needs to be further validated in a larger multicenter cohort in the future. There may be a potential bias due to the circannual or seasonal rhythm of monocytes and lymphocytes (31). On the other hand, since this novel marker is a cost-effective and easily measurable method in the clinics and hospitals, it is expected to be extremely useful as a prognostic marker at the time of initial diagnosis. Besides, due to the correlation between immune cells with antitumor activity and disease prognosis, promoting basic research on the aspects of immunity, mainly monocytes and macrophages, may lead to the development of new therapies.

## Data Availability Statement

The raw data supporting the conclusions of this article will be made available by the authors, without undue reservation.

## Ethics Statement

The studies involving human participants were reviewed and approved by Kobe Children’s Hospital ethics committee. Written informed consent from the participants’ legal guardian/next of kin was not required to participate in this study in accordance with the national legislation and the institutional requirements.

## Author Contributions

AT designed the study methodology, collected data, performed statistical analysis, and wrote the draft. MM designed the study methodology, wrote the draft, and provided valuable discussions. SI, TM, JN, SN, AS, AK, and TI collected data and provided valuable discussions. KS supported writing the draft. DH and YK collected data and supervised the study. All authors have read and approved the final version.

## Conflict of Interest

The authors declare that the research was conducted in the absence of any commercial or financial relationships that could be construed as a potential conflict of interest.

## Abbreviations

ALC: absolute lymphocyte count
AMC: absolute monocyte count
ANC: absolute neutrophil count
CRP: C-reactive protein
Hb: hemoglobin
HR: high-risk
LDH: lactate dehydrogenase
NSE: neuron-specific enolase
OS: overall survival
PFS: progression-free survival
ROC: receiver operating characteristic curve.

## References

[1] Maris JM. Recent advances in neuroblastoma. *N Eng J Med.* (2010) 362:2202–11. 10.1056/NEJMra0804577 20558371 PMC3306838

[2] Pinto NR Applebaum MA Volchenboum SL Matthay KK London WB Ambros PF Advances in risk classification and treatment strategies for neuroblastoma. *J Clin Oncol.* (2015) 33:3008–17. 10.1200/JCO.2014.59.4648 26304901 PMC4567703

[3] Disis ML. Immune regulation of cancer. *J Clin Oncol.* (2010) 28:4531–8. 10.1200/JCO.2009.27.2146 20516428 PMC3041789

[4] Thommen DS Schumacher TN. T cell dysfunction in cancer. *Cancer Cell.* (2018) 33:547–62. 10.1016/j.ccell.2018.03.012 29634943 PMC7116508

[5] Feng F Zheng G Wang Q Liu S Xu G Wang F Low lymphocyte count and high monocyte count predicts poor prognosis of gastric cancer. *BMC Gastroenterol.* (2018) 18:148. 10.1186/s12876-018-0877-9 30305076 PMC6180580

[6] Chan JC Chan DL Diakos CI Engel A Pavlakis N Gill A The lymphocyte-to-monocyte ratio is a superior predictor of overall survival in comparison to established biomarkers of resectable colorectal cancer. *Ann Surg.* (2017) 265:539–46. 10.1097/SLA.0000000000001743 27070934 PMC5300029

[7] Giorgi UD Mego M Scarpi E Giuliano M Giordano A Reuben JM Relationship between lymphocytopenia and circulating tumor cells as prognostic factors for overall survival in metastatic breast cancer. *Clin Breast Cancer.* (2012) 12:264–9. 10.1016/j.clbc.2012.04.004 22591634

[8] Lee SF Luque-Fernandez MA. Prognostic value of lymphocyte-to-monocyte ratio and neutrophil-to-lymphocyte ratio in follicular lymphoma: a retrospective cohort study. *BMJ Open.* (2017) 7:e017904. 10.1136/bmjopen-2017-017904 29101140 PMC5695484

[9] Chan JY Zhang Z Chew W Tan GF Lim CL Zhou L Biological significance and prognostic relevance of peripheral blood neutrophil-to-lymphocyte ratio in soft tissue sarcoma. *Sci Rep.* (2018) 8:11959. 10.1038/s41598-018-30442-5 30097600 PMC6086886

[10] Olingy CE Dinh HQ Hedrick CC. Monocyte heterogeneity and functions in cancer. *J Leukoc Biol.* (2019) 106:309–22. 10.1002/JLB.4RI0818-311R 30776148 PMC6658332

[11] Hanna RN Cekic C Sag D Tacke R Thomas GD Nowyhed H Patrolling monocytes control tumor metastasis to the lung. *Science.* (2015) 350:985–90. 10.1126/science.aac9407 26494174 PMC4869713

[12] Kanda Y. Investigation of the freely available easy-to-use softwa‘e “ZR’ for medical statistics. *Bone Marrow Transplant.* (2013) 48:452–8. 10.1038/bmt.2012.244 23208313 PMC3590441

[13] Weinstein JL Katzenstein HM Cohn SL. Advances in the diagnosis and treatment of neuroblastoma. *Oncologist.* (2003) 8:278–92. 10.1634/theoncologist.8-3-278 12773750

[14] Cohn SL Pearson AD London WB Monclair T Ambros PF Brodeur GM The international neuroblastoma risk group (INRG) classification system: an INRG task force report. *J Clin Oncol.* (2009) 27:289–97. 10.1200/JCO.2008.16.6785 19047291 PMC2650388

[15] Hann HW Evans AE Siegel SE Wong KY Sather H Dalton A Prognostic importance of serum ferritin in patients with stages III and IV neuroblastoma: the childrens cancer study group experience. *Cancer Res.* (1985) 45:2843–8. 3986811

[16] Silber JH Evans AE Fridman M. Models to predict outcome from childhood neuroblastoma: the role of serum ferritin and tumor histology. *Cancer Res.* (1991) 51:1426–33. 1997181

[17] Riley RD Heney D Jones DR Sutton AJ Lambert PC Abrams KR A systematic review of molecular and biological tumor markers in neuroblastoma. *Clin Cancer Res.* (2004) 10:4–12. 10.1158/1078-0432.ccr-1051-2 14734444

[18] Morandi F Barco S Stigliani S Croce M Persico L Lagazio C Altered erythropoiesis and decreased number of erythrocytes in children with neuroblastoma. *Oncotarget.* (2017) 8:53194–209. 10.18632/oncotarget.18285 28881804 PMC5581103

[19] Wang K Shen T Wei S. The CD4/CD8 ratio of tumor-infiltrating lymphocytes at the tumor-host interface has prognostic value in triple-negative breast cancer. *Hum Pathol.* (2017) 69:110–7. 10.1016/j.humpath.2017.09.012 28993275

[20] Tosolini M Kirilovsky A Mlecnik B Fredriksen T Mauger S Bindea G Clinical impact of different classes of infiltrating T cytotoxic and helper cells (Th1, Th2, Treg, Th17) in patients with colorectal cancer. *Cancer Res.* (2011) 71:1263–71. 10.1158/0008-5472.CAN-10-2907 21303976

[21] Lee HL Jang JW Lee SW Yoo SH Kwon JH Nam SW Inflammatory cytokines and change of Th1/Th2 balance as prognostic indicators for hepatocellular carcinoma in patients treated with transarterial chemoembolization. *Sci Rep.* (2019) 9:3260. 10.1038/s41598-019-40078-8 30824840 PMC6397294

[22] Morandi F Pozzi S Barco S Cangemi G Amoroso L Carlini B CD4^+^ CD25^hi^ CD127^–^ Treg and CD4^+^ CD45RO^+^ CD49b^+^ LAG3^+^ Tr1 cells in bone marrow and peripheral blood samples from children with neuroblastoma. *Oncoimmunology.* (2016) 5:e1249553. 10.1080/2162402X.2016.1249553 28123887 PMC5214984

[23] Asgharzadeh S Salo JA Ji L Oberthuer A Fischer M Berthold F Clinical significance of tumor-associated inflammatory cells in metastatic neuroblastoma. *J Clin Oncol.* (2012) 30:3525–32. 10.1200/JCO.2011.40.9169 22927533 PMC3675667

[24] Hashimoto O Yoshida M Koma Y Yanai T Hasegawa D Kosaka Y Collaboration of cancer-associated fibroblasts and tumour-associated macrophages for neuroblastoma development. *J Pathol.* (2016) 240:211–23. 10.1002/path.4769 27425378 PMC5095779

[25] Cassetta L Pollard JW. Targeting macrophages: therapeutic approaches in cancer. *Nat Rev Drug Discov.* (2018) 17:887–904. 10.1038/nrd.2018.169 30361552

[26] Tamura A Hirai H Yokota A Kamio N Sato A Shoji T C/EBPβ is required for survival of Ly6C^–^ monocytes. *Blood.* (2017) 130:1809–18. 10.1182/blood-2017-03-772962 28807982 PMC5649551

[27] Jung K Heishi T Khan OF Kowalski PS Incio J Rahbari NN Ly6C^lo^ monocytes drive immunosuppression and confer resistance to anti-VEGFR2 cancer therapy. *J Clin Invest.* (2017) 127:3039–51. 10.1172/JCI93182 28691930 PMC5531423

[28] Guilliams M Mildner M Yona S. Developmental and functional heterogeneity of monocytes. *Immunity.* (2018) 49:595–613. 10.1016/j.immuni.2018.10.005 30332628

[29] Venstrom JM Zheng J Noor N Danis KE Yeh AW Cheung IY KIR and HLA genotypes are associated with disease progression and survival following autologous hematopoietic stem cell transplantation for high-risk neuroblastoma. *Clin Cancer Res.* (2009) 15:7330–4. 10.1158/1078-0433.CCR-09-1720 19934297 PMC2788079

[30] Matsuno R Toyama D Akiyama K Isoyama K Shiozawa E Yamamoto S. Killer-cell immunoglobulin-like receptor ligand mismatch cord blood transplantation in high-risk neuroblastoma. *Pediatr Int.* (2019) 61:566–71. 10.1111/ped.13861 30974480

[31] Patel AA Yona S. Inherited and environmental factors influence human monocyte heterogeneity. *Front Immunol.* (2019) 10:2581. 10.3389/fimmu.2019.02581 31787976 PMC6854020

